# Lipid Handling Protein Gene Expression in Colorectal Cancer: CD36 and Targeting miRNAs

**DOI:** 10.3390/life12122127

**Published:** 2022-12-16

**Authors:** Andrei Marian Niculae, Maria Dobre, Vlad Herlea, Florina Vasilescu, Laura Cristina Ceafalan, Bogdan Trandafir, Elena Milanesi, Mihail Eugen Hinescu

**Affiliations:** 1 Victor Babes National Institute of Pathology, 050096 Bucharest, Romania; 2 Faculty of Medicine, Carol Davila University of Medicine and Pharmacy, 050474 Bucharest, Romania; 3 Fundeni Clinical Institute, 022328 Bucharest, Romania

**Keywords:** colorectal cancer, lipid metabolism, gene expression, CD36, miR-27a-3p

## Abstract

The reprogramming of lipid metabolism has been highlighted in colorectal cancer (CRC) studies, suggesting a critical role for the scavenger receptor CD36 and fatty acid synthase (FASN) in this malignancy. In this study, we analyzed the gene expression levels of CD36, FASN, the cell surface glypican 4 (GPC4), and the two transporters SLC27A3 and SLC27A4 in 39 paired tumoral and peritumoral tissues from patients with CRC compared with 18 normal colonic mucosae. Moreover, the levels of seven miRNAs targeting CD36 and most of the analyzed genes were evaluated. We found a significant impairment of the expression of all the analyzed genes except GPC4 as well as the differential expression of miR-16-5p, miR-26b-5p, miR-107, miR-195-5p, and miR-27a-3p in the colonic mucosa of CRC patients. Interestingly, CD36 and miR-27a-3p were downregulated and upregulated, respectively, in tumoral tissues compared to peritumoral and control tissues, with a significant negative correlation in the group of patients developing lymph node metastasis. Our results sustain the relationship between CRC and fatty acid metabolism and emphasize the importance of related miRNAs in developing new therapeutic strategies.

## 1. Introduction

Lipid metabolism refers to the synthesis and degradation of lipids, which have a wide range of biological functions. They are important cellular constituents, are involved in energy storage, and participate in cellular functions, including material transport, energy metabolism, and information transfer. Lipids can fuel energy through beta fatty acid oxidation, and fatty acids (FAs), acting as signaling molecules, can control the redox homeostasis, transcriptional, and enzymatic networks and inflammatory response [1]. The reprogramming of the lipid metabolism is one of the hallmarks of cancer [2]. Lipid metabolism dysregulation is involved in cancer initiation and progression, including cell migration, invasion, and angiogenesis [3]. Moreover, it has been suggested to be responsible for the failure of antitumor immunity [4] and has been shown to modulate anticancer drug sensitivity and confer drug resistance [5].

As the second and the third most common cancer in women and men, respectively [6], CRC represents a disease with a major impact on the global health system, with a prediction of 3.2 million new global cases in 2040 [7]. Among the risk factors contributing to CRC onset and progression, lifestyle habits play a prominent role. This disease is associated with the consumption of processed and red meat [1], obesity [2], physical inactivity [3], high alcohol consumption [4], and tobacco use [5]. 

The complexity of the lipid metabolism in CRC has recently been highlighted by an expanding number of new studies that have demonstrated that the lipid metabolism, cellular stress responses, and gut microbiota all work together as important actors in CRC development [6]. FA uptake, a crucial cellular process of lipid metabolism that transports fatty acids for membrane biosynthesis, is involved in energy storage and signaling pathway activation. 

FASN coding for fatty acid synthase, which catalyzes the formation of long-chain fatty acids from acetyl-CoA, malonyl-CoA, and NADPH, plays an important role in the growth and survival of cancers with lipogenic characteristics [7] and has been suggested as a potential therapeutic target for the early stages of CRC [8]. The exogenous uptake of FAs is mediated by specialized transporters, such as CD36 and fatty acid-transporting proteins. CD36 is a membrane glycoprotein receptor with several ligands, including long-chain FA, thrombospondin-1, hexarelin, fibrillar Aβ amyloid peptides, and oxidized low- and high-density lipoproteins (LDL and HDL). It is expressed on the membranes of various cell types [9], with a high expression in the human gastrointestinal tract [10].

Numerous cancers, such as ovarian cancer, glioblastoma, human oral cancers, and breast cancer, have been linked to CD36, which promotes primary tumor growth and disease progression [11,12,13,14]. A recent pan-cancer study revealed that CD36 expression in tumoral tissue and adjacent normal tissues or in cancer cell lines varied, being increased or decreased in tumors according to cancer type [15]. Analyzing the CRC cohort, the study reported reduced levels of CD36 in CRC, suggesting that CD36 appears to play a role in immune infiltration, with its levels positively correlating with infiltrating stromal scores in CRC [15]. A marginally significant prognostic CD36 value, with high expression associated with poorer prognoses, was identified, as previously suggested in a smaller gene expression study on CRC [16]. Other studies have emphasized the critical role of CD36 in promoting the cellular proliferation, progression, and metastasis of primary CRC and other cancers [17,18,19]. Moreover, it has recently been demonstrated that CD36 can regulate glycolysis and tumorigenesis in CRC through the ubiquitination of the cell surface proteoglycan glypican 4 (GPC4). The GPC4 protein level was significantly suppressed in cells with CD36 overexpression, whereas in CD36 knockdown cells, increased levels of GPC4 were found [20].

Along with FASN, CD36, and GPC4, other genes belonging to the FA metabolic process, previously demonstrated to play a role in the progression of different types of cancer, are the two long-chain FA transporters solute carrier family 27 member 3 (SLC27A3) and solute carrier family 27 member 4 (SLC27A4). Although they belong to a family of six related FA transporters, SLC27A3 has demonstrated acyl-CoA synthetase activity, and there is no evidence of its transporter function [21], whereas SLC27A4 is clearly involved in the translocation of long-chain fatty acids across the plasma membrane. No studies have been conducted on their expression in CRC. The role of SLC27A3 in cancer has been investigated only in glioma and lung cancer, evidencing that it was highly expressed in both diseases [22,23]. SLC27A4 is localized intracellularly in intestinal epithelial cells [24] and is the predominant FA transporter present in the intestine that can activate both long- and very long-chain FA [25]. Studies on cancers revealed increased or decreased levels of SLC27A4 according to cancer type [26,27,28,29].

Micro-RNAs (miRNAs) are small noncoding RNA molecules that can bind to target mRNA, inducing the downregulation of the target protein. They are key regulators involved in the initiation, progression, metastasis, and recurrence of CRC [30], and they are promising molecular biomarkers for the diagnosis, prognosis, and therapeutic response in CRC [31,32]. A single miRNA can target several transcripts, affecting the expression of genes involved in one or more pathways. Moreover, miRNAs involved in the regulation of genes belonging to the lipid metabolism appear to be good candidates for therapeutic approaches targeting the lipid metabolism to combat cancer [33].

In this study, we aimed to better understand the involvement of CD36 and the FA metabolism of CRC by analyzing the gene expression levels of CD36, GPC4, FASN, SLC27A3, and SLC27A4 in 39 paired tumoral and peritumoral tissues from patients with CRC compared with 18 normal colonic samples as well as a panel of seven miRNAs targeting CD36 and most of the investigated genes. 

## 2. Materials and Methods

### 2.1. Patients and Collection of Biological Specimens

The investigated cohort comprised 39 patients with primary CRC and 18 cancer-free volunteers (CTRL). All CRC patients were recruited between 2017 and 2021 during tumor surgical resection at the “Fundeni” Clinical Institute in Bucharest, Romania. No patients had received radiotherapy and/or chemotherapy before surgery. For each patient, the tumoral (T) and the corresponding peritumoral (PT) tissues, approximately 8–10 cm from the tumors, were collected and preserved in RNA later for 48–72 h and dry stored at −80 °C until total RNA extraction. RNA from the CTRL cohort was available at the “Victor Babes” National Institute of Pathology biobank, and the exclusion and inclusion criteria are described elsewhere [34]. At the time of sample collection, no control and no patient were under antibiotic treatment.

All individuals signed a written consent form to participate in the study and approved the use of their biological samples according to the Helsinki declaration. The study was approved by the Ethics Committee of the “Victor Babes” National Institute of Pathology (approval number 291 of 8 March 2016, and approval number 78, 3 December 2019) and the Ethics Committee of the “Fundeni” Clinical Institute (11 December 2019). 

### 2.2. Gene Expression Analysis

The total RNA was isolated using the miRNeasy Mini Kit (Qiagen, Hilden, Germany). The reverse transcription of 300 ng of RNA was performed using the RT^2^ First Strand Kit (Qiagen, Hilden, Germany). All these steps were performed according to the manufacturer’s protocols. The expression of CD36, GPC4, FASN, SLC27A3, and SLC27A4 was evaluated using the RT^2^ qPCR Primer Assays PPH01356A, PPH09593A, PPH01012B, PPH20806A, and PPH00471A with RT^2^ SYBR^®^ Green qPCR Mastermix (Qiagen, Hilden, Germany) on an ABI-7500 fast instrument (Thermo Scientific, Waltham, MA, USA). The geometric mean of two reference genes, RPLP0 (PPH21138F) and HPRT1 (PPH01018C), was used to normalize gene expression data.

### 2.3. miRNA Selection and Expression Analysis

Since the literature has suggested several miRNAs targeting CD36 in different tissues, conditions, and diseases, to select a reasonable number of representative miRNAs, we applied the following two-step selection: in silico analysis followed by a literature search of the miRNAs found that were predicted to target CD36 in the bioinformatic analysis. The in silico analysis was performed using DIANA-TarBase v.8, a bioinformatics tool devoted to identifying miRNA–mRNA computed (predicted) or experimentally supported interactions. Using DIANA-TarBase v.8, we first selected a list of miRNAs predicted to target CD36, identifying 13 candidate miRNAs. From this panel, we selected only the miRNAs validated to target CD36 according to the literature’s data. Based on this selection, the expression of the following miRNAs was analyzed: miR-155-5p, miR-16-5p, miR-27a-3p, miR-26b-5p, miR-29a-3p [35], miR-107 [36], and miR-195-5p [37].

The levels of these miRNAs were evaluated in 25 T and corresponding PT tissues and in the whole CTRL cohort (*n* = 18). Ten ng of total RNA were reverse transcribed with the miRCURY LNA RT Kit (Qiagen, GmbH), and the expression levels were evaluated using the miRCURY LNA miRNA PCR Assay (YP02119311, YP00205702, YP00206038, YP00204172, YP00204698, YP00204468, and YP00205869) with the miRCURY LNA SYBR Green PCR Kit. The geometric mean of two reference miRNAs (SNORD38B and SNORD49A) was used for normalization.

### 2.4. In Silico Validation of Gene Expression Results

All transcripts analyzed in this study were investigated in a larger cohort of CRC patients and controls using public databases. To achieve this aim, the OncoDB program (http://oncodb.org/index.html, accessed on 6 June 2022) was used to download and analyze the individual gene expression data generated by RNA-seq from 308 patients with colorectal adenocarcinoma (from the TCGA database) and 41 controls (from the GTEx study).

### 2.5. Immunohistochemistry Analysis

The expression of CD36 in paraffin-embedded tumoral and peritumoral tissues was analyzed by immunohistochemistry using the CD36 antibody NB400-144 (Novus Biologicals, Littleton, CO, USA). Each sample, with a 5-µm thick section, was processed using the BenchMark Ultra Ventana. The deparaffinization of the samples (72 °C for 16 min) was followed by antigen retrieval for 56 min at pH 8 at 100 °C (Ultra CC1). Endogenous peroxidase activity was then blocked for 5 min. The incubation with CD36 primary antibody (36°C for 36 min) was followed by incubation with OptiView HRP Multimer (36 °C, 8 min) and OptiViewDAB (36 °C, 8 min). Counterstaining was carried out with hematoxylin (36 °C, 16 min) and post-counterstaining with bluing reagent (36 °C, 8 min). The slides were examined by a pathologist using a DM750 light microscope (Leica Microsystems GmbH). CD36 expression levels were evaluated by calculating the percentage of positive tumoral cells. The images were acquired using the Axiocam 305 Color (ZEISS).

### 2.6. Statistical Analysis

The statistical analysis for the mRNA and miRNA expression was performed on 2^−ΔCt^ values. The fold change (FC) values (2^−ΔΔCt^) were calculated using different groups as references according to the comparison: FC (T vs. CTRL) = 2^−ΔCt^ T/2^−ΔCt^ CTRL; FC (T vs. PT) = 2^−ΔCt^ T/2^−ΔCt^ PT; and FC (PT vs. CTRL) = 2^−ΔCt^ P T/2^−ΔCt^ CTRL. The results are presented as fold regulation (FR) as follows: when the FC value was above 1 (upregulation), the FR was equal to FC; when the FC value was less than 1 (downregulation), the FR was expressed as the negative inverse of FC. The statistical analysis and generating of graphs were performed using SPSS Version 20.0 and GraphPad Prism 8.4.3, respectively. Differences in sex and age between the patients and controls were tested using the chi-squared test and the *t*-test, respectively. Since the values of the analyzed mRNAs and miRNAs levels were not normally distributed (Shapiro–Wilk test, *p* < 0.05), non-parametric statistical tests were used. The Mann–Whitney test was used to compare mRNAs and miRNAs variations between patients and controls. For the related samples (paired T and PT), the Wilcoxon signed-rank test was applied. The differences in mRNA or miRNA levels between the groups were considered significant when *p* < 0.05.

## 3. Results

The age mean of the control group was 63.28 ± 8.40, and the group comprised 10 females and 8 males. Four controls reported moderated alcohol consumption (22.22%) and one was obese (5.55%). None of the controls had diabetes. Patients and controls were age (*p* = 0.428) and sex-matched (*χ*^2^ = 0.015; *p* = 0.904). Moreover, no differences between the groups were found considering the reported moderate alcohol use (*χ*= 2.868; *p* = 0.090), obesity (*χ*^2^ = 0.729; *p* = 0.393), and diabetes (*χ*^2^ = 1.920; *p* = 0.166). The detailed sociodemographic and clinical characteristics of the CRC patients are displayed in Table 1.

### 3.1. Gene Expression Levels

The expression analysis of four genes belonging to the FA metabolic process (CD36, FASN, SLC27A3, and SLC27A4) and the related gene GPC4 in tumoral (T) CRC tissues compared to the control (CTRL) and peritumoral tissue (PT) revealed a general impairment (shown as FR = fold regulation) of these genes among the groups. In particular, we found the downregulation of CD36, SLC27A3, and SLC27A4 in tumoral tissues compared to the corresponding PT tissues (FR = −5.13, *p* < 0.001; FR = −1.77, *p* < 0.001; and FR = −1.73, *p* < 0.001, respectively), while FASN was upregulated (FR = 1.41, *p* = 0.003). No significant changes in GPC4 levels were observed in this comparison (FR = −1.38, *p* = 0.586). 

When comparing the levels of these genes between T and CTRL tissues, only CD36 and SLC27A4 were downregulated in the tumoral tissue (FR = −3.49, *p* < 0.001 and FR = −1.94, *p* = 0.001, respectively) and FASN was upregulated (FR = 4.20, *p* < 0.001). These results were also validated in a larger cohort of patients and controls using the public OncoDB database (Table 2). 

**Figure 1:**
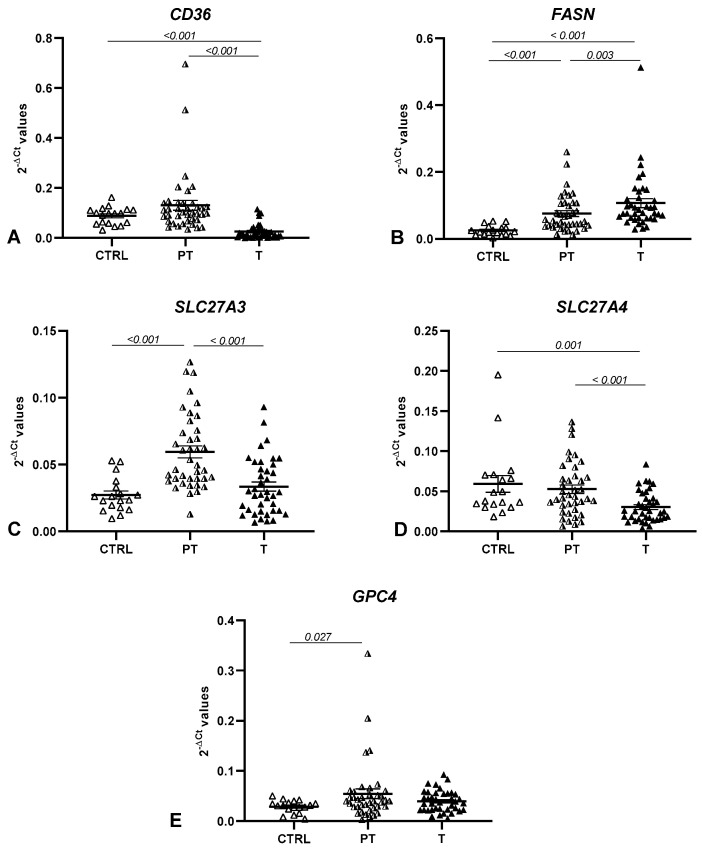
mRNA levels of the analyzed genes in the CTRL, PT, and T tissues. Expression levels are shown as 2^−∆Ct^ values, and horizontal bars represent the expression averages ± the standard error of mean (SEM) of (**A**) the CD36 scavenger receptor; (**B**) FASN fatty acid synthase; (**C**) SLC27A3 solute carrier family 27 member 3; (**D**) SLC27A4 solute carrier family 27 member 4; and (**E**) GPC4 glypican 4. The *p*-value was calculated using the Wilcoxon signed-rank test to compare T vs. PT tissues and using the Mann–Whitney U test to compare T vs. CTRL tissues and PT vs. CTRL tissues.

No changes were observed in CD36 and SLC27A4 mRNA levels in PT compared with CTRL tissues, while SLC27A3, FASN, and GPC4 were upregulated (FR = 2.18, *p* < 0.001; FR = 2.97, *p* < 0.001; and FR = 1.88, *p* = 0.027, respectively; Table 2, Figure 1). 

We did not observe significant changes when comparing the mRNAs levels of the investigated genes in tumoral tissues between patients with lymph node involvement (*n* = 14) and those without lymph node metastasis (*n* = 25).

### 3.2. miRNA Levels

**Figure 2:**
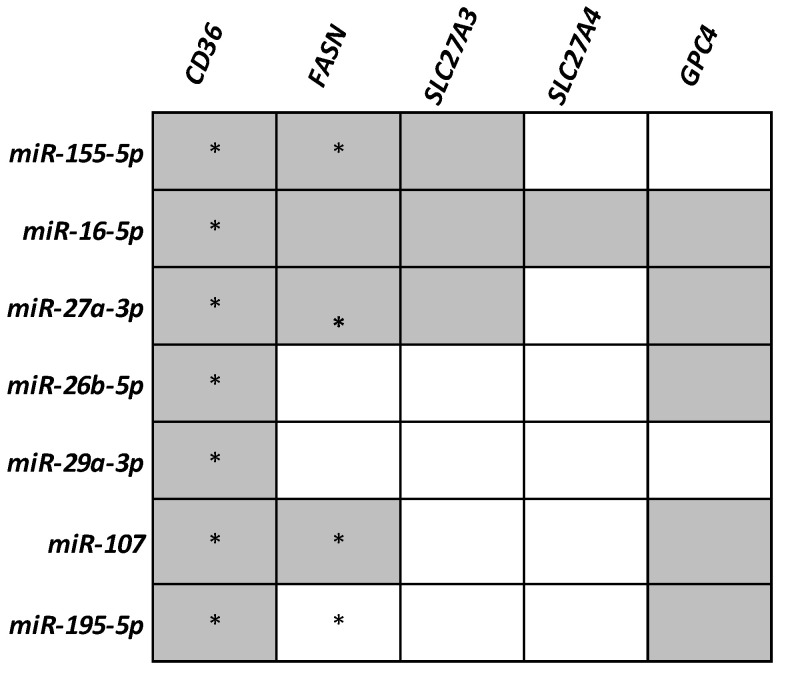
Panel of miRNAs selected for this study. The gray color refers to miRNAs predicted by TarBase v.8, and stars refer to those miRNAs validated to target the analyzed genes.

The in silico miRNA analysis followed by a literature search of the miRNAs found predicted to target CD36 in the bioinformatic analysis resulted in a panel of seven candidate miRNAs. Based on this selection, the expression of the following miRNAs was analyzed: miR-155-5p, miR-16-5p, miR-27a-3p, miR-26b-5p, miR-29a-3p, miR-107, and miR-195-5p. Notably, most selected miRNAs were also suggested to target other genes analyzed in this study (Figure 2).

The qPCR analysis of the seven miRNAs is depicted in Table 3. 

**Figure 3:**
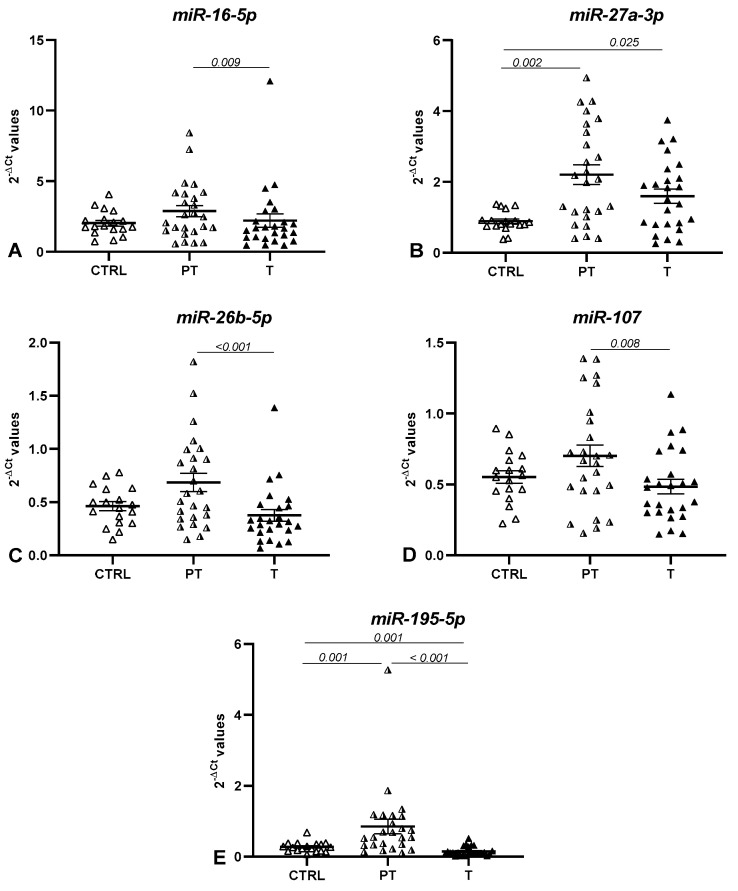
miRNA levels in CTRL, PT, and T tissues. Expression levels are shown as 2^−∆Ct^ values, and horizontal bars represent the expression averages ± the standard error of mean (SEM) of (**A**) miR-16-5p; (**B**) miR-27a-3p; (**C**) miR-26b-5p; (**D**) miR-107; and (**E**) miR-195-5p. (The *p*-value was calculated using the Wilcoxon signed-rank test for comparing T vs. PT tissues and using the Mann–Whitney U test for comparing T vs. CTRL tissues and PT vs. CTRL tissues.

A graphical representation of the miRNAs statistically significant in at least one comparison is reported in Figure 3. 

**Figure 4:**
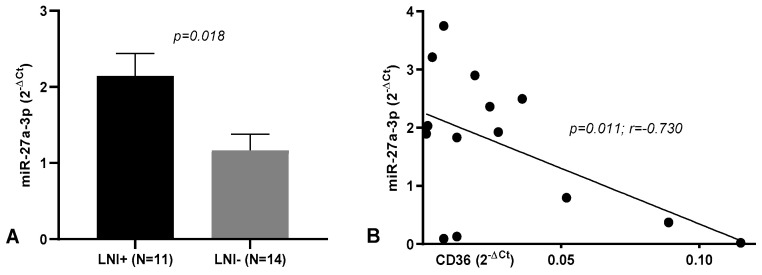
miR-27. a-3p levels in tumoral tissues. (**A**) Comparison between patients presenting lymph node involvement (LNI+) vs. those without (LNI). (**B**) Correlation with CD36 mRNA levels in LNI+ patients.

Notably, when comparing the miRNA levels in tumoral tissues between patients with lymph node involvement (LNI+; *n* = 11) vs. those without lymph node metastasis (LNI-; *n* = 14), we observed that miR-27a-3p was upregulated (*p* = 0.018, FR = 1.84; Figure 4A). No changes were observed when performing the same comparison in PT tissues. This upregulation was also found when comparing patients with perineural invasion (*n* = 6) vs. those without (*n* = 19; *p* = 0.014, FR = 1.75).

### 3.3. miRNA/mRNA Correlations

Pearson correlation analyses, performed in the group of 11 patients presenting lymph nodes involvement, found a negative correlation between CD36 and miR-27a-3p (*p* = 0.011, *r* = −0.730; Figure 4B), which was also reported in the subgroup of 9 patients with lymphovascular invasion (*p* = 0.015, *r* = −0.772). In the same subgroup, a negative correlation was found between SLC27A3 and miR-27a-3p (*p* = 0.006, *r* = −0.825).

### 3.4. CD36 Immunostaining

**Figure 5:**
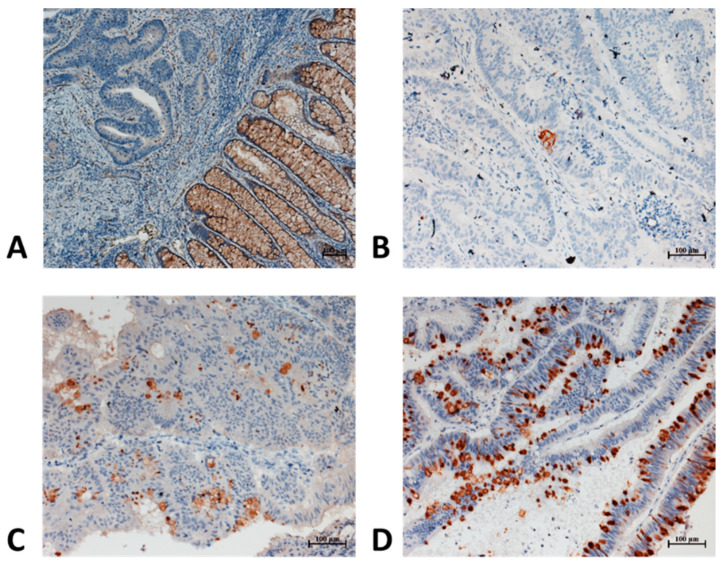
Representative immunohistochemical (IHC) analysis of the expression of CD36 in the tumoral and peritumoral tissues of six samples. (**A**) Tumoral (CD36 negative) vs. normal colon glands (CD36 positive), 100×; (**B**) tumoral cells (CD36 negative) with focal positivity for CD36, 200×; (**C**) and (**D**) tumoral cells with different positivity expression for CD36, 200×.

Subsequently, we investigated CD36 expression in 39 pairs of human colon cancer samples using immunohistochemistry (IHC). CD36 expression was negative in 25 tumor samples examined; weak expression was found in 8 tumor samples, whereas a positive expression was present in 4 tumor samples. In Figure 5, a panel of four representative cases is presented. All peritumoral specimens demonstrated strong CD36 expression in normal colonic glands, endothelial cells, and adipocytes.

## 4. Discussion

In this work, we found a general impairment of the expression of key genes involved in FA metabolism and a panel of miRNAs targeting these transcripts in the colonic mucosa of CRC patients compared with adjacent non-tumoral tissues and normal mucosa. 

The lower expression of CD36 levels found in a recent study comparing 458 CRC tissues with 349 normal tissues [15] was confirmed in our CRC cohort, in which we observed that CD36 levels also were downregulated in comparison with the adjacent normal tissue. Importantly, in line with our results, Fang et al. found that the expression of CD36 sequentially decreases from adenomas to carcinomas, and in vitro and in vivo experiments have shown that this molecule acts as a tumor suppressor. The underpinning mechanism reveals that CD36 is an inhibitor of β-catenin/c-myc-mediated glycolysis through the ubiquitination of glypican-4 (GPC4), leading to the inhibition of downstream aerobic glycolysis [20]. Although a recent study has reported that in CRC, GPC4 is upregulated in tumoral tissues compared with matched adjacent normal mucosa [38], our study only detected upregulation in peritumoral tissue compared to normal mucosa. 

In agreement with our results, which detected increased levels of FASN in T samples compared with both CTRL and PT tissues, other studies have reported FASN overexpression in CRC tissues compared with the controls, both at the transcript and protein levels [39,40]. In addition, its expression was substantially correlated with lymph node metastasis, TNM stage, and poor prognosis [41]. Moreover, an in vitro investigation suggested that cell migration and proliferation were mediated by the FASN/AMPK/mTOR signaling axis, indicating that this pathway is a potential therapeutic target [41]. Another study emphasized the crucial role of FASN in CRC initiation, underlying its importance not only as a therapeutic target for early stages but also as a target for a preventive strategy [8]. Indeed, the use of FA synthase inhibitors for CRC treatment has obtained promising results, both in vitro and in vivo [42,43,44,45,46,47,48].

In our study, we did not find changes in the expression of SLC27A3 in tumoral tissue compared to healthy tissue, which showed the same levels; however, the high expression of this transcript was found in peritumoral tissue. Regarding SLC27A4, no study has investigated its involvement in CRC, and available observations refer to other types of cancers. High levels of SLC27A4 have been detected in bladder cancer [27], renal cell carcinoma [28], and breast cancer [29] compared to controls, whereas uterine cervical cancer has shown reduced levels [26]. Here, for the first time, we proved the mRNA levels of SLC27A4 were also reduced in CRC tumoral tissues compared to both the adjacent peritumoral and the normal colonic tissues. 

In the second part of our work, the expression analysis of a panel of miRNAs targeting CD36 was evaluated. Significantly, the selected miRNAs were also predicted to target many analyzed genes in this study beside CD36. 

Our analysis revealed that three miRNAs, miR-16-5p, miR-26b-5p, and miR-107, reported similar levels of tumoral and normal mucosa, with significant upregulation in peritumoral tissues.

Contrasting results on the expression of miR-16-5p in CRC have been reported in the literature. In line with our findings, many works have found lower levels of this miRNA in tumoral tissues compared to adjacent tissue [49,50,51,52]. Nonetheless, no study has evaluated its expression in normal tissue from independent controls. On the contrary, the significant upregulation of miR-16-5p in CRC tissue compared to adjacent tissue has been observed by Diamantopoulos et al. [53] and confirmed by another research group [54].

miR-26b has been found downregulated in tumoral tissues compared with adjacent normal tissues in CRC patients [55,56,57]. In line with our results, Ma et al. found the downregulation of miR-26b in both colorectal carcinoma and adenoma tissues compared to normal adjacent tissues [58], and miR-26b has been reported to suppress CRC [59,60]. Another study discerned its overexpression in CRC cell lines, where it directly targets and suppresses multiple tumor suppressors. The same study reported upregulated levels in CRC tumor samples from patients with lymphatic metastases and indicated that miR-26b promotes metastasis and could be a therapeutic target for metastatic CRC [61]. 

Another miRNA significantly downregulated in tumoral tissues compared with the peritumoral tissues of the investigated patients was miR-107. The findings regarding the expression of this miRNA in CRC are controversial. Fu et al., whose results were similar to ours, reported the downregulation of miR-107 in CRC patients and colorectal cell lines [62]. Conversely, three other studies indicated increased levels of this miRNA in tumoral samples compared to the controls or adjacent normal tissue [63,64,65]. The discrepancies related to miR-107 expression in CRC could be attributed to disparities in the analyzed tumors in terms of grading, stage, and tumor locations or may simply reflect the great complexity governing intra- and intercellular signaling networks.

In our study, miR-195-5p was found to be downregulated in tumoral CRC tissues compared to control and peritumoral tissues. In several cancers, including CRC, miR-195 functions by targeting the mRNAs of many proteins that act as oncogenes or tumor suppressors. In particular, in CRC, this miRNA targets genes involved in apoptosis, viability, metastasis, proliferation, migration, and radio- and chemotherapy resistance [66,67]. In line with the findings of our work, other studies have reported that miR-195 expression significantly decreased in CRC specimens compared with adjacent normal tissues [67,68,69] or control tissues [70]. Moreover, a bioinformatics study on colon adenocarcinoma data from the TCGA database found that patients with decreased levels of miR-195-5p in tumor tissues experienced significantly shorter survival [71].

The current study also revealed that miR-27a-3p was upregulated in peritumoral tissues and tumoral tissues compared to control. Interestingly, its levels were higher in the tumoral tissues of CRC patients with lymph node metastasis. In addition, miR-27a has been indicated to be an oncogene or tumor suppressor in several types of cancer [72]. In CRC tissues, its levels have been found to increase gradually from normal colonic mucosa to adenoma and adenocarcinoma, demonstrating its role as an onco-miRNA in CRC [73]. Other studies on tissues and serum have found miR-27a-3p overexpression in CRC compared to healthy controls [74,75]. Moreover, in vitro studies have reported that its levels correlate with a high capacity for proliferation, invasion, and migration in various tumors [73,76,77] including CRC, where it regulates apoptosis and the cell cycle [78]. The negative correlation between miR-27a and CD36 expression in CRC tissues has been reported for the first time in this work. A direct interaction between CD36 and miR-27a-3p levels in CRC has not been proven. However, interesting results were obtained by Kim et al., who found that, in 3T3-L1 adipocytes retrovirally infected with miR-27a, the CD36 expression level was repressed by miR-27a overexpression [79]. Corroborating this observation, Zhang et al. demonstrated that a miR-27a/b inhibitor significantly elevated CD36 mRNA and protein expression, while an miR-27a/b mimic repressed CD36 levels in THP-1 macrophages [80].

Last, in this study, we observed that miR-27a-3p levels negatively correlated with CD36 mRNA levels in tumoral tissues in the group of patients reporting lymph node metastasis. Similar findings were observed in a study that analyzed miRNA levels in the plasma of CRC patients and found that the relative expression levels of miR-27a in patients with lymph node metastasis and distant metastasis were higher than those in patients without lymph node metastasis and distant metastasis [81], suggesting a potential diagnostic and prognostic value for miR-27a.

Interestingly, certain miRNAs analyzed in this study targeting FA metabolism genes have been shown to be involved in chemotherapy resistance in CRC. This is the case for miR-27a-3p, miR-107, and miR-195-5p. The high expression of miR-27a-3p in two independent cohorts of CRC patients was associated with increased resistance to chemotherapy, which was later confirmed in a CRC cell model system [82]. In addition, miR-107 has been found to induce chemoresistance in CRC through the CAB39-AMPK-mTOR pathway [83], and miR-195-5p has been found to reduce CRC cell stemness and chemoresistance [84]. These findings suggest that the miRNAs involved in the FA metabolism could also be promising targets for overcoming chemoresistance and ameliorating standard therapeutic approaches to CRC.

## 5. Conclusions

The results of this study highlight the involvement of CD36 and a panel of genes belonging to the FA metabolism as well as their targeting miRNAs in CRC. In particular, we emphasize the connection between CD36 and miR-27a-3p, providing an observation of their negative correlation in CRC patients with lymph node invasion, suggesting a potential diagnostic and prognostic value for miR-27a-3p. However, the lack of an in vitro assay proving the direct or indirect correlation between the presence of lymph node metastasis, CD36, and miR-27a-3p expression levels represents a limitation of this study. In conclusion, our results sustain the relationship between CRC and FA metabolism, which could lead to the development of new therapeutic strategies for this malignancy, which acts on gene and miRNAs expression.

## Figures and Tables

**Table 1:** Sociodemographic and clinical characteristics of the patients involved in the study.

Features	CRC Patients (*n* = 39)
Age (mean ± SD)	65.62 ± 11.00
Sex	21F; 18M
Moderate alcohol use *	46.6% Yes; 53.4% No
Obesity *	13.3% Yes; 86.7% No
Diabetes *	10% Yes; 90% No
Tumor Characteristics	
Localization	Ascending colon (*n* = 5) Transverse colon (*n* = 5) Descending colon (*n* = 5) Sigmoid (*n* = 9) RSJ (*n* = 9) Rectum (*n* = 6)
Grade	G1 (*n* = 6) G2 (*n* = 26) G3 (*n* = 7)
TNM (T = size of the primary tumor; *N* = degree of spread to regional lymph nodes; M = presence of distant metastasis)	T2 N0 M0 (*n* = 4); T2 N1 M0 (*n* = 1); T2 N2 M0 (*n* = 1); T3 N0 M0 (*n* = 16); T3 N0 M1 (*n* = 1); T3 N1 M0 (*n* = 7); T3 N2 M0 (*n* = 4); T4 N0 M0 (*n* = 3); T4 N1 M0 (*n* = 1); T4 N2 M0 (*n* = 1);
Lymphovascular invasion	Yes (*n* = 10); No (*n* = 29)
Perineural invasion	Yes (*n* = 6); No (*n* = 33)
Tumoral Markers	
CEA—carcinoembryonic antigen (ng/mL) mean (min–max) (*n* = 26)	28.08 (0.65–444)
CA 19-9—carbohydrate antigen (U/mL) mean (min–max) (*n* = 26)	35.58 (3.47–291.25)
AFP—alpha-fetoprotein (ng/mL) mean (min–max) (*n* = 20)	3.70 (0.60–11.84)
Biochemical Parameters	
Hemoglobin (g/dL) mean (min–max) (*n* = 36)	12.37 (6.90–17.90)
WBC—white blood cells (N/µL) mean (min–max) (*n* = 36)	6377 (1016–14840)
Platelets (N103/µL) mean (min–max) (*n* = 36)	303 (152–777)
INR—international normalized ratio mean (min–max) (*n* = 36)	1.04 (0.87–1.58)
Fibrinogen (mg/dL) mean (min–max) (*n* = 32)	426.58 (138–796)
Albumin (g/dL) mean (min–max) (*n* = 31)	3.93 (2.30–5.48)

* Data available for 30 CRC patients.

**Table 2:** Genes differentially expressed among the groups (*p* < 0.05).

Genes	39 T vs. 39 PT		39 PT vs. 18 CTRL		39 T vs. 18 CTRL		308 T vs. 41 CTRL (OncoDB)	
	*p*-Value *	FR	*p*-Value **	FR	*p*-Value **	FR	*p*-Value ^$^	FR
CD36	<0.001	−5.13	0.223	1.47	<0.001	−3.49	<0.001	−3.79
FASN	0.003	1.41	<0.001	2.97	<0.001	4.2	<0.001	2.53
SLC27A3	<0.001	−1.77	<0.001	2.18	0.46	1.23	0.015	−1.16
SLC27A4	<0.001	−1.73	0.81	−1.12	0.001	−1.94	<0.001	−1.77
GPC4	0.586	−1.38	0.027	1.88	0.107	1.37	<0.001	1.63

* Wilcoxon signed-rank test. ** Mann–Whitney test. ^$^ Student’s *t*-test. FR = fold regulation.

**Table 3:** miRNAs differentially expressed among groups (*p* < 0.05).

miRNAs	T vs. PT		T vs. CTRL		PT vs. CTRL	
	*p*-Value *	FR	*p*-Value **	FR	*p*-Value **	FR
miR-155-5p	0.339	−1.24	0.127	−1.14	0.768	1.09
miR-16-5p	0.009	−1.31	0.290	1.09	0.313	1.42
miR-27a-3p	0.065	−1.38	0.025	1.79	0.002	2.48
miR-26b-5p	<0.001	−1.83	0.052	−1.23	0.127	1.48
miR-29a-3p	0.737	1.11	0.140	1.38	0.389	1.24
miR-107	0.008	−1.45	0.200	−1.14	0.257	1.27
miR-195-5p	<0.001	−5.78	0.001	−1.86	0.001	3.10

* Wilcoxon signed-rank test. ** Mann–Whitney test.

## Data Availability

The data presented in this study are available on reasonable request from the corresponding author.
